# The causal effect of folate on major depressive disorder: Mendelian randomization, LDSC, and colocalization analysis

**DOI:** 10.1097/MD.0000000000046149

**Published:** 2025-11-21

**Authors:** Dele Liu, Fengxiang Liao, Yanping Xiong, Zizhen Huang

**Affiliations:** a Jiangxi Provincial People’s Hospital, The First Affiliated Hospital of Nanchang Medical College, Nanchang, Jiangxi, China.

**Keywords:** colocalization analysis, DNA methylation, folate, LDSC, major depression, Mendelian randomization

## Abstract

Dietary intervention is beneficial in reducing the risk of major depressive disorder (MDD). Nevertheless, whether the intake of macronutrients and vitamin B supplements is causally associated with MDD remains unclear. Univariate Mendelian randomization was performed to identify the causal effect of the relative intake of macronutrients (including fat, protein, and carbohydrates) and vitamin B (including vitamin B6, vitamin B12, and folate) on MDD in 338,111 individuals. Moreover, we further analyzed the genetic associations between the intake of macronutrients and vitamin B and the progression of MDD via linkage disequilibrium score regression and colocalization. Additionally, we explored the influence of DNA methylation caused by folate supplementation on the risk of MDD. Our univariate Mendelian randomization findings revealed that high folate levels were associated with a low risk of MDD (OR = 0.010, 95% CI: 0.001–0.184). Moreover, the relative intake of protein, fat, and carbohydrate, along with vitamin B6 and vitamin B12, was not associated with MDD. Linkage disequilibrium score regression revealed a genetic correlation between folate supplementation and MDD (rg = −0.470, *P* = .001). Colocalization analysis revealed that folate supplementation and MDD share a causal variant (rs115879259). Additionally, 3 folate-related CpG sites (cg03651886, cg03986574, and cg10664184) were causally associated with MDD progression. Our study provides evidence that folate supplementation decreases the risk of MDD.

## 1. Introduction

Major depressive disorder (MDD), a prevalent and severe psychiatric condition, significantly increases economic burden.^[1]^ Emerging evidence indicates that both genetic predispositions and environmental factors influence MDD.^[2–4]^ Although 828 antidepressants are under development, many have been discontinued or no progress has been made.^[5]^ Recently, increasing evidence has shown that dietary habits are associated with the pathogenesis and progression of depression.^[6]^ Thus, identifying an appropriate dietary pattern is beneficial for reducing MDD progression.

Several studies have suggested that macronutrients and vitamin B may reduce the risk for MDD. A case-control study including 130 patients revealed that poor diet in patients with depression was related to depression.^[7]^ A Finnish population-based study suggested that vitamin B may be associated with the risk of MDD.^[8]^ However, due to immeasurable and uncontrollable confounding factors, the correlations among vitamin B, macronutrients and MDD remain to be identified.

Recently, statistical methods have been used to identify the causal effects of the risk factors on this disease. Linkage disequilibrium score regression (LDSC) using GWAS summary dataset can be used to evaluate genetic correlations.^[9]^ Mendelian randomization (MR) is an epidemic statistical technique used to further calculate this causation without measurement bias, confounding bias, or reverse causality interference.^[10]^ Colocalization analysis was used to further assess whether exposures and outcomes shared causal variants.^[11]^

Accordingly, this study was to clarify whether the intake of macronutrients and vitamin B (including folate, vitamin B6, and B12) are causally associated with MDD. We also aimed to examine their genetic correlations with MDD and to explore the potential role of folate-induced DNA methylation in the development of MDD.

## 2. Materials and methods

### 2.1. Design overview

**Figure 1.**
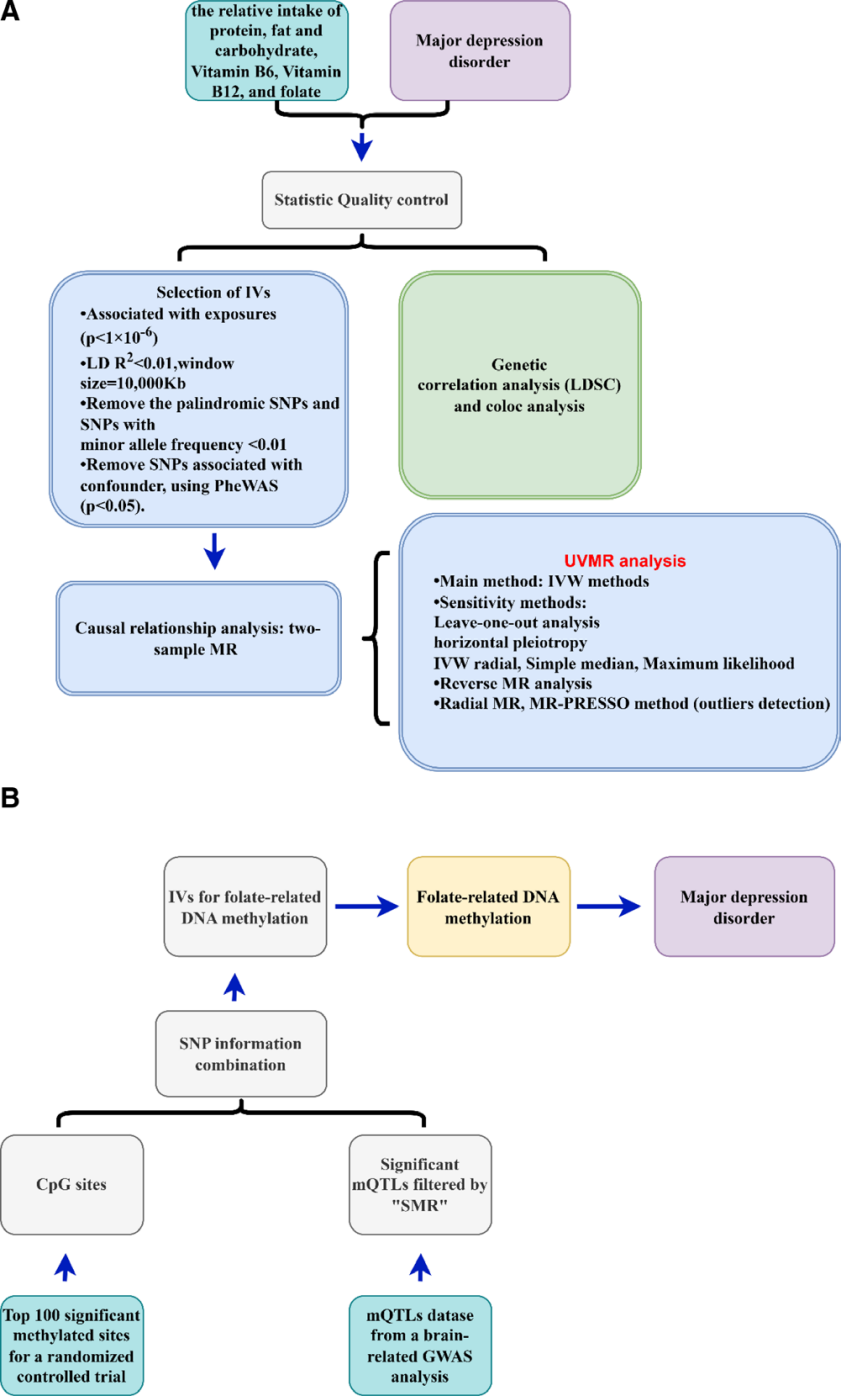
Main design of this study. (A) The UVMR, LDSC and colocalization analysis was used to detect the genetic and causal correlation between the relative intake of macronutrient or vitamin B and MDD. (B) Analysis flow to identify the causal relationship between folate-related DNA methylation and MDD. IVs = instrumental variables, IVW = inverse variance weighted, LDSC = linkage disequilibrium score regression, MDD = major depressive disorder, SMR = summary-based Mendelian randomization, SNPs = single-nucleotide polymorphisms, UVMR = univariate Mendelian randomization.

Figure 1 shows an outline of the overall design. First, we used univariate Mendelian randomization (UVMR) to analyze the associations between the relative intake of protein, carbohydrate, and fat and supplementation with vitamin B6, vitamin B12, folate, and vitamin C. Three supplemental MR methods and several sensitivity methods were used to improve the robustness and reliability of the results. LDSC was performed to estimate the genetic correlation between risk factors and MDD. Colocalization was conducted to determine the probability that the 2 traits share the same causal variation. We also investigated the relationship between DNA methylation caused by folate supplementation and MDD, as shown in Figure 1B.

### 2.2. GWAS resource for exposures

A GWAS dataset concerning the relative intake of protein, fat, and carbohydrates was obtained from a GWAS with 235,000 individuals of European ancestry.^[12]^ Notably, this study was performed for relative macronutrient intake, correcting for total energy intake, which made it possible to discuss the effects of different proportions of macronutrient intake on MDD.^[13]^ Single-nucleotide polymorphisms (SNPs) associated with B vitamins were extracted from a UKBB, which explained 0.62%, 1.14% and 1.19% of the variance in folate, vitamin B6 and vitamin B12, respectively.^[14]^

### 2.3. GWAS resource for outcomes

The GWAS summary dataset for MDD was obtained from the FinnGen study with 38,225 cases and 299,886 controls.^[15]^ MDD is defined as a mood disorder with a clinical course involving 1 or more episodes of serious psychological depression that last 2 or more weeks each, do not have intervening episodes of mania or hypomania, and are characterized by a loss of interest or pleasure in almost all activities and by some or all disturbances in appetite, sleep, or psychomotor functioning; a decrease in energy; difficulties in thinking or making decisions; loss of self-esteem or feelings of guilt; and suicidal thoughts or attempts.

### 2.4. Instrumental variables (IVs) selection

IVs were based on 3 types of consumption: IVs were strongly associated with the relative intake of protein, fat, and carbohydrates. No association between IVs and any potential confounding factors was required. IVs affect the risk of diabetic nephropathy through the relative intake of protein, fat, and carbohydrates. At the same time, IVs with *P* < 5 × 10^−8^ and a minor allele frequency > 0.01 were selected. Furthermore, we calculated the F statistic to avoid weak instrument bias using the formula $F\text{-}statistic={\left(\frac{Beta}{SE}\right)}^{2}.$^[16]^ The F-statistics of the weak IVs were <10. We obtained 6 SNPs related to relative protein intake, 6 SNPs associated with relative fat intake, and 12 SNPs associated with carbohydrate intake. Phenotypes significantly associated with SNPs are shown in ST-1, Supplemental Digital Content, https://links.lww.com/MD/Q751.

### 2.5. LDSC analysis

We estimated the genetic correction (rg) between relative macronutrient intake and MDD using LDSC, without potential bias due to sample overlap.^[17]^ GWAS summary statistics were processed based on 1000 genomes. SNPs that were strand ambiguous, repeated, and had a minor allele frequency < 0.01 were excluded.

### 2.6. Colocalization analysis

The probability was evaluated using 5 hypotheses.^[18]^ The gene was classified as having evidence of colocalization based on the posterior probability of Hypothesis 4 > 90%.

### 2.7. Two-sample MR: folate-related DNA methylation and MDD

A randomized controlled trial including 87 participants investigated significantly methylated positions after supplementation with folate.^[19]^ We considered only the top 100 differentially methylated positions (Benjamini–Hochberg-adjusted *P* value < .05). Moreover, the significant mQTLs (*P* < 1 × 10^−7^) were extracted from a brain-related GWAS meta-analysis via “SMR” software.^[20]^ To assess the causal effect of DNA methylation at folate-related CpGs in MDD, we performed two-sample MR. First, we looked up the identified mQTLs based on the top 100 CpG positions, and only 9 CpGs were found, as depicted in Figure 1B. We subsequently extracted GWAS summary data for each SNP corresponding to a CpG. Finally, we combined information on SNP–MDD associations with information on the SNP-methylation associations and performed MR analysis, as shown in ST-6, Supplemental Digital Content, https://links.lww.com/MD/Q751.

### 2.8. Statistical analysis

Inverse variance weighting (IVW) is the main method used in MR analysis, and the weighted median, penalized weighted median and simple median are supplementary methods to IVW. When there is no pleiotropy, IVW is prioritized as the primary method.^[21]^ Heterogeneity was assessed using Cochrane *Q* value and corrected using the random effects model IVW.^[22]^ The MR-Egger intercept method was used to evaluate the uncorrelated horizontal pleiotropy of the SNPs.^[23]^ Specifically, outliers were corrected for horizontal pleiotropy by them. Leave-one-out analysis was performed to validate the reliance on MR results.^[24]^ Statistical significance of the causal relationship was defined as an FDR < 0.05. *R*^2^, which explains the variance of the SNP, was calculated as $\;\;R^{2}=\frac{F\text{-}statistic}{n-2+F\text{-}statistic}.$

UVMR and MVMR were performed via the R packages “TwoSampleMR and “MendelianRandomization.”^[24,25]^ MR-PRESSO was conducted by “MRPRESSO.”^[22]^ LDSC and colocalization analyses were performed via the “coloc” and “ldscr” packages.^[26,27]^ These analyses were performed using R software version 4.1.2 (https://www.r-project.org/). “SMR” software was used to extract the significant mQTL sites.^[28]^

## 3. Results

### 3.1. The UVMR analysis of the relative macronutrient intake, vitamin B, and MDD

**Figure 2.**
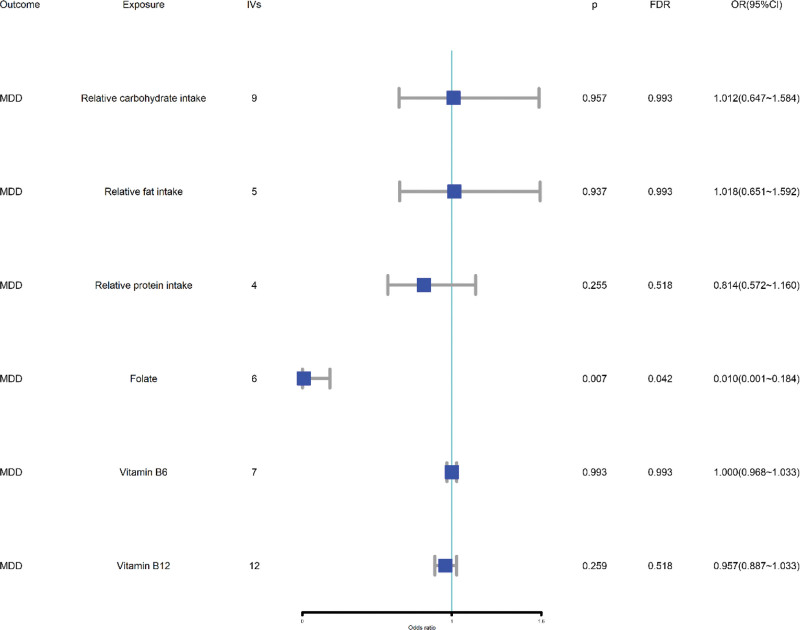
The IVW values for MR. IVs that actually participate in the MR study, after harmonizing the information from exposure and outcome. 95% LCI = lower limit of 95% CI, 95% UCI = upper limit of 95% CI, FDR = false discovery rate, IVs = instrumental variables, IVW = inverse variance weighted, MDD = major depressive disorder, MR = Mendelian randomization.

The UVMR results revealed that a 1-unit higher log odds ratio decreased the standard deviation of patients with 0.01 (OR = 0.010, 95% CI = 0.001–0.184). A steiger filtering analysis confirmed this cause (p_steiger_test_ = 8.023E−08). There was no evidence of a causal association between the relative intake of protein, fat, carbohydrates, and vitamins B6 or B12 (all *P* > .05), as depicted in Figure 2. Pleiotropy and heterogeneity were not detected in most of the MR results shown in ST-3, Supplemental Digital Content, https://links.lww.com/MD/Q751, except for carbohydrates. The leave-one-out analysis suggested that these results were affected by outlier SNPs (shown in SF-1, Supplemental Digital Content, https://links.lww.com/MD/Q751). To test the stability of the MR results, IVW radial, simple median, and maximum likelihood methods were used in the MR analyses. The associations estimated via IVW were consistent with the results obtained via the other 3 methods, as depicted in Figure 2, ST-3 and SF-2, Supplemental Digital Content, https://links.lww.com/MD/Q751. Radial MR and MR-PRESSO analyses revealed only one outlier in the IVs of carbohydrate intake. After removing the outlier SNPs, the results still supported no association between the relative intake of carbohydrates and MDD, as shown in ST-4 and SF-3, Supplemental Digital Content, https://links.lww.com/MD/Q751. Furthermore, we conducted a reverse MR analysis to ensure that the causal direction was not from MDD to folate levels. The results showed that MDD did not affect folate levels, as demonstrated by ST-5, Supplemental Digital Content, https://links.lww.com/MD/Q751.

### 3.2. The genetic correlation analysis and colocalization analysis

**Figure 3.**
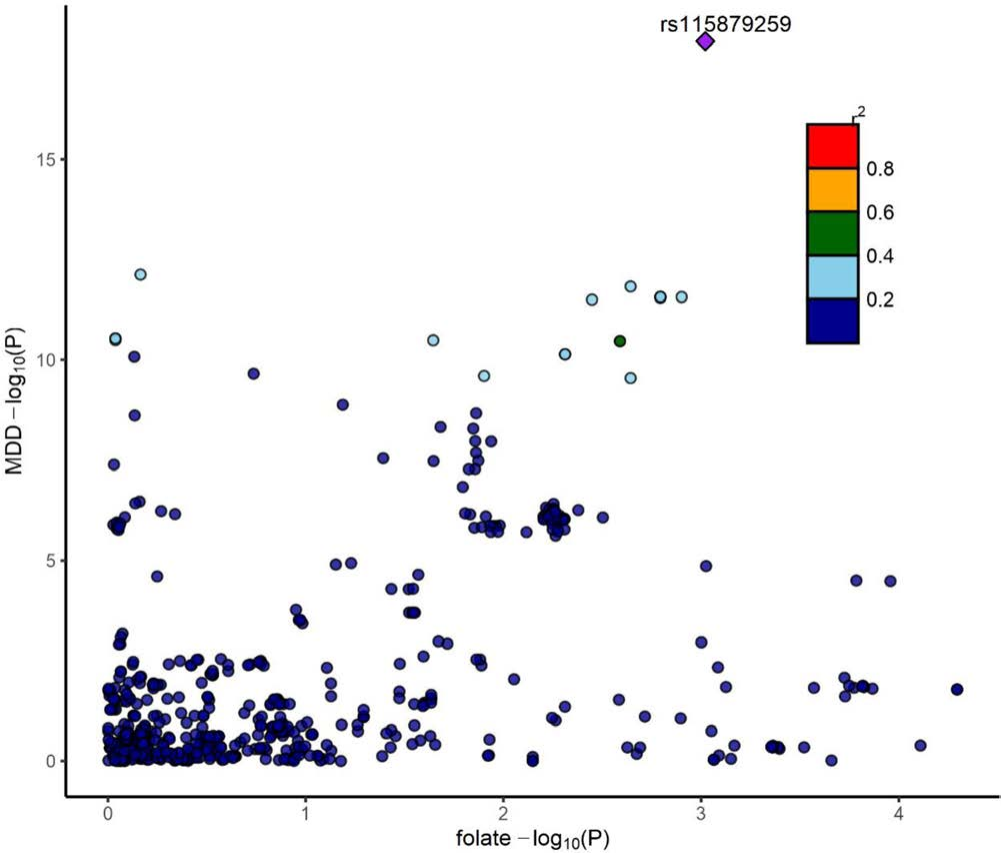
Locus comparison for the shared causal variant for the associations of folate supplementation and MDD in the gene region (Chr6:32109239–33109239), which is located within ± 500Kb from rs12722072. MDD = major depressive disorder.

We performed LDSC regression and colocalization analyses to estimate the genetic correlation between the relative intake of macronutrients and vitamin B. The LDSC results revealed a potential negative correlation between folate levels and MDD (r_g_ = −0.470, *P* = .001) but no genetic association with other factors, as shown in Table 1. Moreover, colocalization analysis was conducted to further identify whether folate supplementation and MDD shared a common genetic causal variant in 9 gene regions. We found that supplementation with folate and MDD had a 91.8% PP.H4 shared a causal variant (rs115879259), as depicted in Figure 3 and ST-6, Supplemental Digital Content, https://links.lww.com/MD/Q751.

**Table 1:** LDSC analysis results.

Trait 1	Trait 2	Genetic correlation (r_g_)	*P*
Relative carbohydrate intake	MDD	−0.042	.286
Relative fat intake	MDD	0.009	.836
Relative protein intake	MDD	−0.043	.336
Folate	MDD	−0.47	.001
Vitamin B12	MDD	0.044	.713
Vitamin B6	MDD	−0.055	.619

LDSC = linkage disequilibrium score regression, MDD = major depressive disorder.

### 3.3. The folate-related DNA methylation and MDD

**Figure 4.**
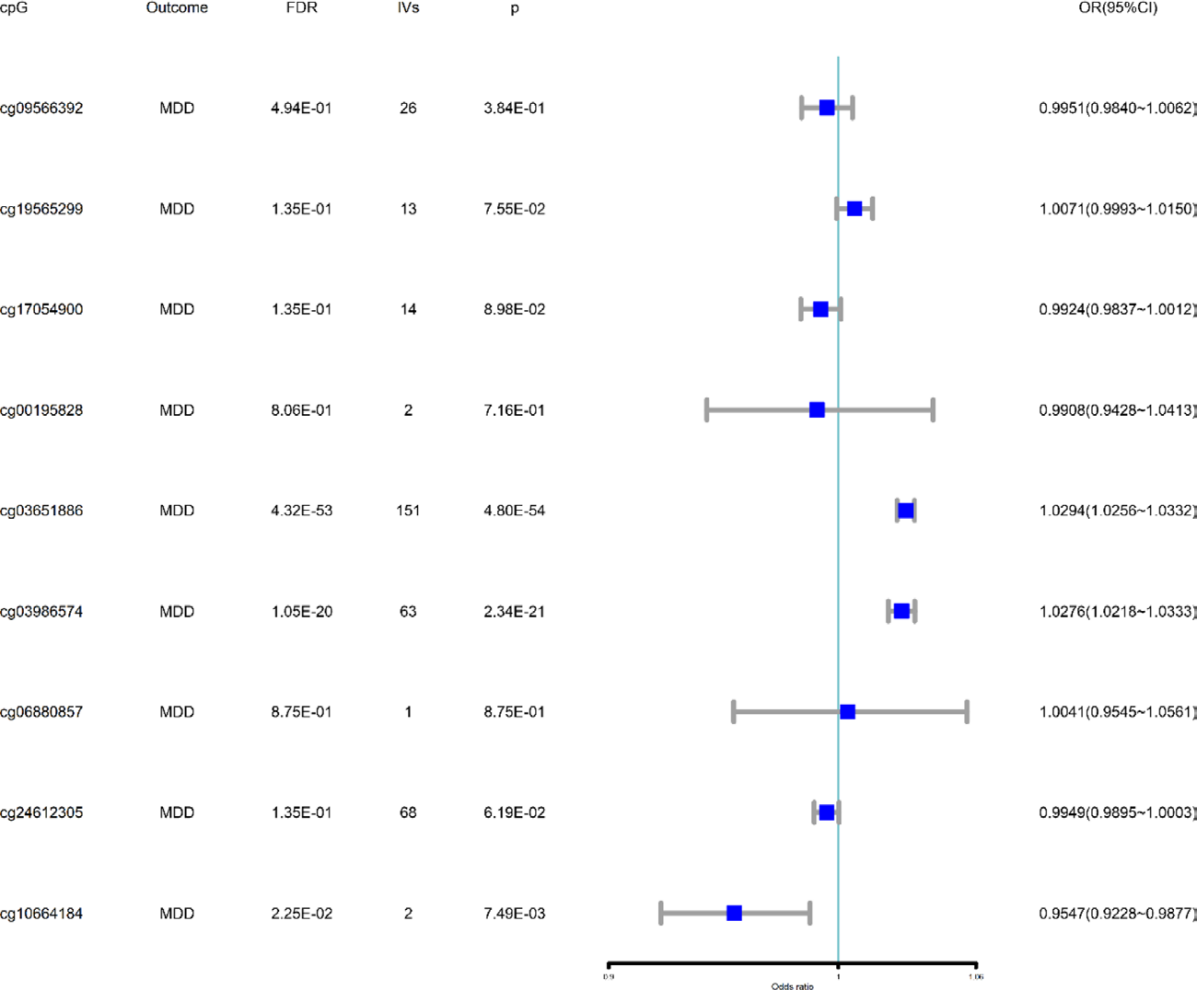
The IVW values for folate-related CpG-MDD MR results. FDR = false discovery rate, IVs = instrumental variables, IVW = inverse variance weighted, MDD = major depressive disorder, MR = Mendelian randomization.

To assess the causal effect of DNA methylation, such as folate-related CpG positions, on MDD, we identified significant mQTLs in the brain-related GWAS summary dataset and performed MR analysis. We found that cg03651886 and SF1, Supplemental Digital Content, https://links.lww.com/MD/Q751 methylation (cg03986574) had a positive causal effect on MDD (FDR < 0.01), whereas DDA1 methylation (cg10664184) was negatively related to MDD (FDR < 0.01), as depicted in Figure 4 and ST-8, Supplemental Digital Content, https://links.lww.com/MD/Q751.

## 4. Discussion

MDD is a complex mental disorder resulting from the combined effects of many factors, including environmental, genetic, psychological, and nutritional factors.^[29]^ Owing to the substantial heritability of MDD, understanding it from a genetic architecture perspective has increasingly received increasing attention. Several GWASs have identified many variants and estimated a heritability of 8.4 for MDD.^[3,30]^ SNP rs6295 was found to be related to an increased risk of MDD through the suppression of 5-HT1A transcription.^[31]^ Moreover, a GWAS meta-analysis provided several lines of evidence that rs4680 in COMT, is a risk variant for MDD.^[32]^ In addition, LDSC analysis has revealed that MDD is genetically correlated with other mental disorders.^[33]^ However, little is known about the genetic correlation between macronutrient intake, vitamin B intake and MDD. Our LDSC analysis revealed that folate supplementation had a negative genetic correlation with MDD. Furthermore, we found that rs12722072 was the shared variant for folate supplementation and MDD. rs12722072 is a missense variant located in the gene region of HLA-DQA1. HLA-DQA1 is pivotal in the immune system and contains peptides derived from extracellular proteins. Several studies have suggested that individuals with higher levels of inflammatory cytokines are more likely to develop MDD. Thus, folate supplementation may benefit patients with MDD by alleviating immune disorders.^[34]^

Emerging research on nutritional psychiatry suggests that nutritional factors also play decisive roles in MDD.^[35]^ Several epidemiological studies have demonstrated that vitamin B levels may be correlated with depression.^[36–41]^ Folate, an essential form of vitamin B, provides sufficient 1-carbon unit substrates for normal metabolic processes in the body.^[42]^ Folate deficiency can cause abnormal synthesis of purine and thymidine precursors, downregulation of DNA methylation, and increased homocysteine levels.^[43]^ A Rotterdam study revealed that depressed individuals were more likely to develop folate deficiency and hyperhomocysteinemia, and another study identified the predictive value of folate for MDD.^[44,45]^ Surprisingly, a clinical study of 2524 adults revealed no significant relationship between folate levels and depression symptom scores.^[46]^ These inconsistent results may be attributed to potential confounding factors. Our UVMR results revealed that increased folate levels were associated with a 0.01-fold lower risk of MDD, which is consistent with the findings of previous studies.^[8,47]^ A possible mechanism may be that folate deficiency disturbs mitochondrial function and normal metabolism.^[48]^ Moreover, some studies have shown that individuals with lower levels of vitamins B6 and B12 are more likely to have MDD.^[45,49]^ However, other studies found no evidence after controlling for confounders.^[46]^ Our UVMR results did not reveal that any association between vitamin B6 or B12 and MDD.

Emerging evidence suggests that epigenetic regulation plays a vital role in psychiatric disorders and could explain the biological effects of environmental factors on MDD.^[50]^ Variable DNA methylation sites have been found to be involved in the progression of MDD. A clinical study revealed that hypomethylation of synapsins is related to the upregulation of MDD-related genes.^[51]^ Moreover, increased methylation of rs7294919 is positively correlated with the risk of MDD.^[52]^ Here, we aimed to explore the effects of DNA methylation resulting from folate supplementation on the risk of MDD. Our results revealed that 3 CpG sites (cg03651886, cg03986574, and cg10664184) are causally associated with MDD. These sites were annotated to genes involved in several biological pathways, such as ubiquitination degradation, hydrolase activity, and pre-mRNA splicing.

Macronutrients, the primary source of energy for the body, include protein, fat, and carbohydrates. The appropriate pattern of macronutrient intake in individuals with MDD has also been studied extensively. A ketogenic diet with high-fat, moderate-protein, and low-carbohydrate intake is considered beneficial for MDD through supplementation with GABA.^[53]^ A cross-sectional study revealed a U-shaped relationship between fat intake and MDD indicating that only a moderate dose of fat could decrease the risk of MDD.^[54]^ In addition, several studies have focused on the effects of specific types of fats on MDD. Trans unsaturated fatty acids are associated with the progression of MDD, whereas polyunsaturated fatty acid intake may be beneficial for MDD by maintaining neuronal homeostasis.^[55,56]^ Although our results indicated that there was no causal relationship between fat intake and MDD, a potential nonlinear relationship needs to be explored using individual datasets. A ketogenic diet suggests that its lower intake is more conducive to fat consumption and, thus, to the production of ketogens. A cross-sectional study based on a public dataset suggested that low carbohydrate intake decreased the risk of depression, whereas a randomized controlled trial demonstrated no significant association.^[57,58]^ Our MR results also revealed no association between the relative carbohydrate intake and MDD. Furthermore, evidence of the relationship between protein intake and MDD is limited. A study of a national cohort indicated that increased protein intake had a protective effect on MDD only in men.^[59]^ However, our results did not indicate that the relative protein intake could affect MDD. Proteins are also sources of amino acids. A NHANES study showed that tryptophan intake was negatively correlated with MDD.^[60]^

However, our study has several limitations. First, our MR study was designed to examine linear effects on MDD. Therefore, more individual-level data are necessary for nonlinear MR, as there may be potential nonlinear relationships. Second, the source of protein (such as fish or milk protein) and the type of fatty acid need to be further explored.^[61]^ Third, only individuals of European descent were included in our study, and the reliability of causal associations should be verified in other non-European populations. Fourth, horizontal pleiotropy, which leads to strong false positives, is common in MR analysis.^[23,62]^ Nevertheless, existing methods make it easy to detect uncorrelated pleiotropy, whereas correlated pleiotropy, another type of horizontal pleiotropy, remains a challenging problem.^[63–65]^ Therefore, although horizontal pleiotropy was not detected in these MR analyses using the horizontal pleiotropy test, correlated horizontal pleiotropy may still exist.

## Acknowledgments

The authors thank all investigators and participants from the FinnGen consortium and the UKBB. We also thank all investigators who contributed to the GWASs of the risk factors.

## Author contributions

**Conceptualization:** Dele Liu.

**Data curation:** Dele Liu, Zizhen Huang.

**Methodology:** Dele Liu.

**Project administration:** Fengxiang Liao, Yanping Xiong, Zizhen Huang.

**Validation:** Zizhen Huang.

**Writing – original draft:** Dele Liu.

**Writing – review & editing:** Zizhen Huang.

## Supplementary Material


