# Supplementary material for: The causal effect of folate on major depressive disorder: Mendelian randomization, LDSC, and colocalization analysis
Source: Medicine (Baltimore). 2025 Nov 21;104(47):e46149. doi: 10.1097/MD.0000000000046149 (PMC12643625; doi:10.1097/MD.0000000000046149)
Supplement: Supplementary file 1 [file medi-104-e46149-s001.pdf]

Exposure	SNP	EA	NEA	MAF	Beta	SE	p.value	F	R^2
Relative carbohydrate intake	rs10206338	A	G	0.57	-0.02	0.00	0.00	31.95	1.77%
	rs10433500	A	G	0.63	0.02	0.00	0.00	31.61	
	rs10510554	T	C	0.44	0.02	0.00	0.00	28.44	
	rs10962121	T	G	0.48	-0.02	0.00	0.00	28.44	
	rs1104608	C	G	0.42	0.02	0.00	0.00	40.11	
	rs2472297	T	C	0.21	-0.02	0.00	0.00	25.00	
	rs36123991	T	G	0.19	0.02	0.00	0.00	36.00	
	rs4420638	A	G	0.82	-0.02	0.00	0.00	48.80	
	rs7012637	A	G	0.49	0.02	0.00	0.00	30.51	
	rs7190396	T	G	0.60	0.02	0.00	0.00	33.32	
	rs8097672	A	T	0.85	0.02	0.00	0.00	38.91	
	rs838144	T	C	0.53	-0.02	0.00	0.00	71.36	
Relative fat intake	rs1229984	T	C	0.05	0.10	0.01	0.00	121.77	1.36%
	rs10468280	A	G	0.61	-0.02	0.00	0.00	58.78	
	rs33988101	T	G	0.50	-0.03	0.00	0.00	113.47	
	rs57193069	A	G	0.55	-0.02	0.00	0.00	31.60	
	rs7012814	A	G	0.49	-0.02	0.00	0.00	46.22	
Relative protein intake	rs13146907	A	G	0.63	-0.02	0.00	0.00	59.46	1.23%
	rs1461729	A	G	0.10	0.03	0.01	0.00	48.09	
	rs1603978	A	C	0.69	0.02	0.00	0.00	41.11	
	rs445551	A	G	0.32	0.02	0.00	0.00	32.04	
	rs838133	A	G	0.43	-0.03	0.00	0.00	111.75	
Folate	rs114513083	A	G	0.05	0.00	0.00	0.00	21.74	1.19%
	rs12722072	G	A	0.19	0.00	0.00	0.00	88.58	
	rs34015603	C	T	0.42	0.00	0.00	0.00	21.39	
	rs10882704	T	G	0.10	0.00	0.00	0.00	23.87	
	rs10771098	A	G	0.12	0.00	0.00	0.00	31.43	
	rs77843149	C	T	0.02	0.01	0.00	0.00	25.68	
	rs73206023	G	A	0.01	0.01	0.00	0.00	21.82	
	rs1873144	A	T	0.12	0.00	0.00	0.00	24.74	
	rs533793605	A	C	0.18	0.00	0.00	0.00	21.42	
Vitamin B6	rs6672805	T	C	0.08	0.01	0.00	0.00	21.54	0.62%
	rs7545529	G	A	0.32	0.00	0.00	0.00	21.83	
	rs141382571	C	T	0.01	0.02	0.00	0.00	21.64	
	rs138463872	C	G	0.00	0.04	0.01	0.00	28.47	
	rs146838262	A	G	0.00	0.04	0.01	0.00	22.64	

	rs62117263	A	G	0.01	0.02	0.00	0.00	24.29	
	rs145449541	G	A	0.01	0.03	0.00	0.00	37.78	
	rs6769778	C	A	0.20	0.01	0.00	0.00	20.90	
	rs28523286	T	C	0.26	0.00	0.00	0.00	21.84	
	rs116311939	G	A	0.00	0.03	0.01	0.00	21.88	
	rs73327924	T	C	0.05	0.01	0.00	0.00	24.93	
	rs343014	G	T	0.02	0.02	0.00	0.00	22.91	
	rs190599873	G	A	0.01	0.02	0.00	0.00	28.36	
	rs148743408	A	C	0.02	0.01	0.00	0.00	21.28	
	rs7960595	A	T	0.47	0.00	0.00	0.00	21.80	
	rs12866096	T	C	0.02	0.02	0.00	0.00	22.55	
	rs4772328	A	G	0.02	0.02	0.00	0.00	22.35	
	rs117361416	C	T	0.01	0.02	0.00	0.00	20.86	
	rs116017697	T	A	0.04	0.01	0.00	0.00	23.32	
Vitamin B12	rs12025246	C	G	0.02	0.17	0.04	0.00	21.01	1.14%
	rs114554135	G	A	0.01	0.21	0.05	0.00	21.24	
	rs79324875	C	T	0.04	-0.12	0.03	0.00	24.88	
	rs6458690	G	A	0.37	-0.07	0.01	0.00	44.75	
	rs1801222	A	G	0.36	-0.09	0.01	0.00	74.66	
	rs12780845	G	A	0.32	0.07	0.01	0.00	37.84	
	rs526934	G	A	0.28	-0.08	0.01	0.00	56.09	
	rs61960878	A	G	0.09	0.09	0.02	0.00	24.13	
	rs67792343	C	T	0.17	-0.06	0.01	0.00	21.29	
	rs2972578	G	A	0.22	-0.06	0.01	0.00	26.19	
	rs506897	G	C	0.48	-0.11	0.01	0.00	115.74	
	rs148225761	G	T	0.01	0.33	0.07	0.00	23.57	
	rs1131603	C	T	0.05	0.13	0.02	0.00	30.06	

ST-1: the IVs of exposures. EA, effect allele. NEA, non-effect allele. MAF, minor allele frequency. F, F-statistic, is valued by (Beta/SE)^2.

SNP	PheWAS phenotype	p.value
rs2472297	Memory loss	0.0001284
rs2472297	Rotator cuff (capsule) sprain	0.004629
rs2472297	Acquired hypothyroidism	0.00478
rs2472297	Cardiac arrhythmia NOS	0.004931
rs2472297	Syncope and collapse	0.005614
rs2472297	Cardiac dysrhythmias	0.005655
rs2472297	Other specified cardiac dysrhythmias	0.006004
rs2472297	Fracture of pelvis	0.00624
rs2472297	Anxiety, phobic and dissociative disorders	0.007178
rs2472297	Abnormal findings on study of brain, nervous system	0.008094
rs2472297	Postmenopausal bleeding	0.008496
rs2472297	Corneal degenerations	0.009176
rs2472297	Epiphora	0.009232
rs2472297	Generalized hyperhidrosis	0.009431
rs2472297	Esophageal atresia/tracheoesophageal fistula	0.009626
rs2472297	Heart valve disorders	0.009812
rs2472297	Disturbances of sulphur-bearing amino-acid metabolism	0.01082
rs2472297	Obstruction of bile duct	0.01097
rs2472297	Inflammatory diseases of female pelvic organs	0.01098
rs2472297	Inflammatory disease of cervix, vagina, and vulva	0.01114
rs2472297	Abnormal involuntary movements	0.01309
rs2472297	Eustachian tube disorders	0.01342
rs2472297	Conduct disorders	0.01395
rs2472297	Adverse effects of adrenal cortical steroids	0.0143
rs2472297	Acute laryngitis and tracheitis	0.01453
rs2472297	Paranoid disorders	0.0146
rs2472297	Posttraumatic stress disorder	0.01478
rs2472297	Other hemoglobinopathies	0.01535
rs2472297	Anxiety disorder	0.01541
rs2472297	Other disorders of back	0.01547
rs2472297	Appendicitis	0.01621
rs2472297	progressive myopia	0.01639
rs2472297	Schizophrenia	0.01649
rs2472297	Sialoadenitis	0.01656
rs2472297	Malaise and fatigue	0.01691
rs2472297	Disturbances of amino-acid transport	0.0176

rs2472297	Other disorders of soft tissues	0.01771
rs2472297	Bladder neck obstruction	0.01806
rs2472297	Keratitis	0.01821
rs2472297	Neurological disorders due to brain damage	0.01951
rs2472297	Skin neoplasm of uncertain behavior	0.02038
rs2472297	Male genital disorders	0.02044
rs2472297	Respiratory abnormalities	0.02045
rs2472297	Otitis media	0.02098
rs2472297	Facial nerve disorders	0.02184
rs2472297	Mild cognitive impairment	0.02197
rs2472297	Urethral hypermobility/ISD	0.02333
rs2472297	Hemorrhage of gastrointestinal tract	0.02418
rs2472297	Secondary malignant neoplasm of liver	0.02428
rs2472297	Precordial pain	0.02439
rs2472297	Inflammation of eyelids	0.02443
rs2472297	Chronic sinusitis	0.02452
rs2472297	Disorders of refraction and accommodation	0.02486
rs2472297	Disease of capillaries	0.02593
rs2472297	Suicidal ideation or attempt	0.02744
rs2472297	Astigmatism	0.02812
rs2472297	Wheezing and painful respiration	0.02878
rs2472297	Cervicitis and endocervicitis	0.02899
rs2472297	Disorders of coccyx	0.02972
rs2472297	Chronic renal failure	0.03051
rs2472297	Spasm of muscle	0.03123
rs2472297	Disorders of sweat glands	0.03141
rs2472297	Pancreatic cancer	0.03178
rs2472297	Chorioretinal scars	0.03214
rs2472297	Magnesium metabolism disorder	0.03303
rs2472297	Epilepsy, recurrent seizures, convulsions	0.03334
rs2472297	Painful respiration	0.03371
rs2472297	Appendiceal conditions	0.034
rs2472297	Other specified gastritis	0.03443
rs2472297	Colon cancer	0.03447
rs2472297	Acute pharyngitis	0.03539
rs2472297	Disorders of lacrimal system	0.03559
rs2472297	Phosphorus metabolism disorder	0.03565

rs2472297	Tobacco use disorder	0.03662
rs2472297	Osteitis deformans and osteopathies associated with other disorders	0.03785
rs2472297	Psoriatic arthropathy	0.03816
rs2472297	Convulsions	0.03833
rs2472297	Pallor and flushing	0.03854
rs2472297	Ankylosis of joint	0.04009
rs2472297	Systemic lupus erythematosus	0.04204
rs2472297	Hidradenitis	0.04255
rs2472297	Other disorders of bladder	0.04265
rs2472297	Other biliary tract disease	0.04312
rs2472297	Clotting factor deficiency	0.04323
rs2472297	Insulin pump user	0.04434
rs2472297	Toxic multinodular goiter	0.04445
rs2472297	Viral hepatitis	0.04451
rs2472297	Purpura and other hemorrhagic conditions	0.04476
rs2472297	Degeneration of intervertebral disc	0.04502
rs2472297	Cholangitis	0.04514
rs2472297	Abnormal serum enzyme levels	0.04523
rs2472297	Congenital coagulation defects	0.04616
rs2472297	Other and unspecified disc disorder	0.04637
rs2472297	Personality disorders	0.04903
rs2472297	Retinal drusen	0.04907
rs2472297	Discoid lupus erythematosus	0.04947

ST-2: the significantly association between IVs with phenotype by PheWAS. The significantly association is defined by p.value<0.05.

Outcome	Exposure	Methods	IVs	p.value	OR	95%LCI	95%UCI	$p_{heterogeneity}$	$p_{pleiotropy}$
**Depression**	**Relative carbohydrate intake**	**IVW radial**	**9**	**0.957**	**1.012**	**0.647**	**1.584**	**0.039**	**0.191**
		**Simple median**	**9**	**0.657**	**1.111**	**0.699**	**1.766**		
		**Maximum likelihood**	**9**	**0.937**	**1.013**	**0.734**	**1.398**		
**Depression**	**Relative fat intake**	**IVW radial**	**5**	**0.937**	**1.018**	**0.651**	**1.592**	**0.227**	**0.199**
		**Simple median**	**5**	**0.370**	**0.767**	**0.430**	**1.369**		
		**Maximum likelihood**	**5**	**0.924**	**1.019**	**0.696**	**1.490**		
**Depression**	**Relative protein intake**	**IVW radial**	**NA**	**NA**	**NA**	**NA**	**NA**	**0.608**	**0.481**
		**Simple median**	**4**	**0.506**	**0.863**	**0.558**	**1.333**		
		**Maximum likelihood**	**4**	**0.255**	**0.813**	**0.569**	**1.161**		
**Depression**	**Folate**	**IVW radial**	**6**	**0.002**	**0.0021**	**0.0001**	**0.113**	**0.575**	**0.370**
		**Simple median**	**6**	**0.022**	**0.0009**	**0.0002**	**0.355**		
		**Maximum likelihood**	**6**	**0.009**	**0.0019**	**0.0001**	**0.210**		
**Depression**	**Vitamin B6**	**IVW radial**	**7**	**0.992**	**0.9999**	**0.9725**	**1.028**	**0.618**	**0.189**
		**Simple median**	**7**	**0.772**	**0.9932**	**0.9480**	**1.041**		
		**Maximum likelihood**	**7**	**0.993**	**0.9999**	**0.9677**	**1.033**		
**Depression**	**Vitamin B12**	**IVW radial**	**12**	**0.165**	**0.957**	**0.899**	**1.018**	**0.779**	**0.432**
		**Simple median**	**12**	**0.638**	**0.975**	**0.876**	**1.085**		
		**Maximum likelihood**	**12**	**0.260**	**0.957**	**0.886**	**1.033**		

ST-3: Mendelian randomization results by IVW radial, weight median, Simple median and Maximum likelihood. 95%LCI, the lower limit of 95% CI; 95%UCI, the upper limit of 95% CI.

Outcome	Exposure	No. of SNPs	No. of removing SNPs*	IVW			No. of removing SNPs*	MR-PRESSO			
				OR	*P*-value	Q *P*-value		Global test *P*-value	Distortion test *P*-value	OR	*P*-value
MDD	Relative carbohydrate intake	12	1	1.012	0.957	0.038	1	0.036	0.569	1.27008	1.40E-01
MDD	Relative fat intake	5	0	1.018	0.937	0.226	0	0.296	NA	1.01182	9.41E-01
MDD	Relative protein intake	5	NA	NA	NA	NA	0	0.677	NA	0.81142	2.41E-01
MDD	Folate	9	0	0.002	0.002	0.588	0	0.702	NA	0.9999	2.72E-02
MDD	Vitamin B6	19	0	0.9999	0.992	0.618	0	0.624	NA	1.8664	9.93E-01
MDD	Vitamin B12	13	0	0.957	0.164	0.781	0	0.812	NA	0.9610	1.71E-01

ST-4. we performed Radial MR analyses using modified second-order weights to identify outliers. inverse variance weighted (IVW); single nucleotide polymorphism (SNP). Cochran's Q statistic in IVW methods to assess heterogeneity of the Wald estimates across variants, P-value < 0.05 indicates the presence of heterogeneity. MR-PRESSO Global test detected horizontal pleiotropy and MR-PRESSO distortion test detected whether statistically significant differences in the causal estimates before and after correction for outliers. If the P values of both the global and distortion tests were less than 0.05, which indicates the existence of horizontal pleiotropy, the outlier-adjusted causal estimates for relationships were presented.

Outcome	Exposure	Methods	IVs	p.value	OR	95%LCI	95%UCI	$p_{heterogeneity}$	$p_{pleiotropy}$
MDD	Folate	Inverse variance weighted	8	5.42E-01	0.999	0.994	1.003	0.595	0.981
		IVW radial	8	4.93E-01	0.999	0.994	1.003		
		Simple median	8	3.02E-01	0.997	0.991	1.003		
		Maximum likelihood	8	5.38E-01	0.999	0.994	1.003		

ST-5: Mendelian randomization results by reverse MR study. 95%LCI, the lower limit of 95% CI; 95%UCI, the upper limit of 95% CI.

Trait 1	Region	SNP	Chromosome	Position	N_SNPs	PP.H0	PP.H1	PP.H2	PP.H3	PP.H4
Folate	Chr3:154169204-155169204	rs114513083	3	154669204	3419	54.60%	23.50%	9.20%	3.95%	0.08%
Folate	Chr6:32109239-33109239	rs12722072	6	32609239	786	38.24%	6.79%	79.95%	1.40%	91.80%
Folate	Chr8:136193328-137193328	rs34015603	8	136693328	3907	48.69%	32.61%	6.40%	4.29%	8.02%
Folate	Chr10:97253645-98253645	rs10882704	10	97753645	3910	48.62%	33.25%	6.69%	4.57%	6.87%
Folate	Chr12:24239554-25239554	rs10771098	12	24739554	4072	0.41%	60.44%	0.19%	28.67%	10.29%
Folate	Chr12:95328915-96328915	rs77843149	12	95828915	4218	22.57%	47.11%	3.51%	7.32%	19.50%
Folate	Chr12:117076080-118076080	rs73206023	12	117576080	3599	45.29%	23.04%	6.60%	3.35%	21.72%
Folate	Chr15:62713296-63713296	rs1873144	15	63213296	3841	16.02%	63.19%	2.86%	11.26%	6.67%
Folate	Chr17:70793138-71793138	rs533793605	17	71293138	4086	75.89%	4.90%	12.42%	0.80%	5.97%

ST-6. the result of colocalization analysis. PP, posterior probability. PP.H0= Posterior probability for Hypothesis 0 (no causal variants) in the colocalization analysis. PP.H1= Posterior probability for Hypothesis 1 (causal variant for sleep trait) in the colocalization analysis. PP.H2= Posterior probability for Hypothesis 2 (causal variant for LTL) in the colocalization analysis. PP.H3= Posterior probability for Hypothesis 3 (distinct causal variants) in the colocalization analysis. PP.H4= Posterior probability for Hypothesis 4 (shared causal variants) in the colocalization analysis.

cpG	SNP	Chr	BP	A1	A2	EAF	Beta	SE	p.value	F
cg09566392	rs12617097	2.00	11277441	A	G	0.75	-0.33	0.05	1.091E-10	41.65
cg09566392	rs72785431	2.00	11279449	G	A	0.74	-0.29	0.05	1.138E-08	32.59
cg09566392	rs7605804	2.00	11280069	C	T	0.75	-0.32	0.05	5.500E-10	38.49
cg09566392	rs4668714	2.00	11281689	C	T	0.76	-0.32	0.05	3.116E-10	39.60
cg09566392	rs35536828	2.00	11283005	C	T	0.76	-0.32	0.05	2.338E-10	40.16
cg09566392	rs34722769	2.00	11287492	C	T	0.78	-0.35	0.05	8.304E-11	42.18
cg09566392	rs12994920	2.00	11288306	C	T	0.76	-0.34	0.05	5.769E-11	42.90
cg09566392	rs12995564	2.00	11288577	C	T	0.76	-0.36	0.05	5.580E-12	47.47
cg09566392	rs13000987	2.00	11289612	G	T	0.76	-0.36	0.05	6.138E-12	47.29
cg09566392	rs71439048	2.00	11290584	G	A	0.76	-0.36	0.05	5.860E-12	47.38
cg09566392	rs34333866	2.00	11290622	C	T	0.76	-0.35	0.05	1.075E-11	46.19
cg09566392	rs6744659	2.00	11291337	T	C	0.76	-0.35	0.05	3.993E-11	43.62
cg09566392	rs6754718	2.00	11291348	G	T	0.76	-0.35	0.05	3.993E-11	43.62
cg09566392	rs6754745	2.00	11291399	G	A	0.76	-0.35	0.05	3.993E-11	43.62
cg09566392	rs10929723	2.00	11291447	C	T	0.76	-0.35	0.05	3.993E-11	43.62
cg09566392	rs10929724	2.00	11291671	A	G	0.76	-0.35	0.05	3.993E-11	43.62
cg09566392	rs12612929	2.00	11291910	A	C	0.76	-0.35	0.05	3.967E-11	43.63
cg09566392	rs34344767	2.00	11292051	C	T	0.76	-0.35	0.05	3.985E-11	43.62
cg09566392	rs13022766	2.00	11292187	C	G	0.76	-0.35	0.05	4.227E-11	43.51
cg09566392	rs4669695	2.00	11292605	T	C	0.76	-0.35	0.05	2.113E-11	44.86
cg09566392	rs13028675	2.00	11293451	G	A	0.77	-0.35	0.05	2.777E-11	44.33
cg09566392	rs13030385	2.00	11293504	C	T	0.77	-0.36	0.05	2.267E-11	44.73
cg09566392	rs13031027	2.00	11293791	C	T	0.77	-0.35	0.05	3.156E-11	44.08
cg09566392	rs34315339	2.00	11293845	G	A	0.77	-0.35	0.05	3.156E-11	44.08
cg09566392	rs12615623	2.00	11294890	A	C	0.77	-0.36	0.05	1.837E-11	45.14
cg09566392	rs7601782	2.00	11296492	G	C	0.77	-0.44	0.08	9.927E-09	32.86
cg19565299	rs62192027	2.00	242697106	C	T	0.12	-0.91	0.15	2.359E-09	35.65
cg19565299	rs34290285	2.00	242698640	A	G	0.27	-0.66	0.11	6.251E-09	33.76
cg19565299	rs35305862	2.00	242700770	G	A	0.12	-0.97	0.15	4.828E-11	43.25
cg19565299	rs2002092	2.00	242701647	T	C	0.12	-0.95	0.15	1.796E-10	40.68
cg19565299	rs4449174	2.00	242702538	G	C	0.12	-0.95	0.15	1.796E-10	40.68
cg19565299	rs62192037	2.00	242702812	A	G	0.12	-0.95	0.15	1.796E-10	40.68
cg19565299	rs146871826	2.00	242703317	T	C	0.12	-0.95	0.15	1.796E-10	40.68
cg19565299	rs6754066	2.00	242703363	A	G	0.12	-0.97	0.15	6.121E-11	42.78
cg19565299	rs5839764	2.00	242703618	G	C	0.38	-0.61	0.10	3.425E-09	34.93
cg19565299	rs77984138	2.00	242703937	A	G	0.12	-0.92	0.15	1.282E-09	36.84

cg19565299	rs6739113	2.00	242706956	T	C	0.12	-0.92	0.15	1.282E-09	36.84
cg19565299	rs6707874	2.00	242709363	G	A	0.38	-0.65	0.10	1.155E-10	41.54
cg19565299	rs7582691	2.00	242710197	T	C	0.43	-0.60	0.10	2.889E-09	35.26
cg19565299	rs62192043	2.00	242711282	A	G	0.27	-0.68	0.11	1.078E-09	37.18
cg19565299	rs62193062	2.00	242728009	G	A	0.18	-0.78	0.13	1.316E-09	36.79
cg17054900	rs242117	3.00	154026467	C	T	0.86	0.67	0.11	3.417E-10	39.42
cg17054900	rs355778	3.00	154030349	T	C	0.86	0.67	0.11	3.417E-10	39.42
cg17054900	rs355776	3.00	154035626	A	T	0.14	-0.67	0.11	3.417E-10	39.42
cg17054900	rs355774	3.00	154036196	G	A	0.86	0.67	0.11	3.417E-10	39.42
cg17054900	rs355773	3.00	154036657	T	C	0.86	0.67	0.11	3.417E-10	39.42
cg17054900	rs355770	3.00	154040273	A	G	0.86	0.67	0.11	3.417E-10	39.42
cg17054900	rs355769	3.00	154042550	C	T	0.86	0.67	0.11	3.417E-10	39.42
cg17054900	rs355768	3.00	154045001	G	A	0.86	0.61	0.11	1.565E-08	31.97
cg17054900	rs355767	3.00	154045895	A	G	0.86	0.66	0.11	7.283E-10	37.94
cg17054900	rs701147	3.00	154048295	T	C	0.86	0.66	0.11	7.283E-10	37.94
cg17054900	rs701145	3.00	154054799	C	T	0.86	0.66	0.11	9.328E-10	37.46
cg17054900	rs789294	3.00	154057741	G	C	0.86	0.66	0.11	9.328E-10	37.46
cg17054900	rs701144	3.00	154059576	T	C	0.86	0.66	0.11	9.328E-10	37.46
cg17054900	rs701143	3.00	154060047	G	A	0.86	0.66	0.11	9.328E-10	37.46
cg17054900	rs701141	3.00	154061053	A	G	0.86	0.66	0.11	9.328E-10	37.46
cg00195828	rs2409092	8.00	8682192	T	A	0.43	0.24	0.04	5.863E-08	29.41
cg00195828	rs12545499	8.00	8682878	C	T	0.44	0.24	0.04	5.406E-08	29.57
cg00195828	rs7013471	8.00	8687325	A	G	0.47	0.24	0.04	6.940E-08	29.08
cg03651886	rs11002554	10.00	80203825	G	A	0.84	-0.60	0.08	2.691E-13	53.42
cg03651886	rs11002555	10.00	80203900	G	T	0.85	-0.60	0.08	2.457E-13	53.60
cg03651886	rs11002556	10.00	80204152	A	C	0.85	-0.60	0.08	2.779E-13	53.36
cg03651886	rs11002557	10.00	80204681	C	T	0.85	-0.60	0.08	2.779E-13	53.36
cg03651886	rs11002558	10.00	80205413	A	G	0.85	-0.60	0.08	2.779E-13	53.36
cg03651886	rs987814	10.00	80206689	A	C	0.53	0.42	0.06	5.072E-12	47.66
cg03651886	rs10824609	10.00	80206903	G	A	0.85	-0.61	0.08	1.530E-13	54.53
cg03651886	rs2670195	10.00	80207281	G	T	0.53	0.42	0.06	5.072E-12	47.66
cg03651886	rs12784427	10.00	80207497	T	G	0.94	-1.18	0.13	1.661E-19	81.61
cg03651886	rs11002559	10.00	80207743	C	A	0.85	-0.61	0.08	7.815E-14	55.85
cg03651886	rs737150	10.00	80207846	T	G	0.53	0.42	0.06	5.072E-12	47.66
cg03651886	rs11002560	10.00	80208565	G	T	0.85	-0.61	0.08	7.815E-14	55.85
cg03651886	rs2670197	10.00	80208671	C	T	0.53	0.42	0.06	5.072E-12	47.66
cg03651886	rs2248789	10.00	80208772	C	T	0.53	0.42	0.06	5.072E-12	47.66

cg03651886	rs34195866	10.00	80210199	C	T	0.94	-1.14	0.13	1.030E-18	78.00
cg03651886	rs919889	10.00	80211193	C	T	0.53	0.42	0.06	6.761E-12	47.10
cg03651886	rs60368868	10.00	80211695	C	T	0.94	-1.18	0.13	1.661E-19	81.61
cg03651886	rs34854197	10.00	80212318	C	G	0.95	-1.18	0.14	2.268E-18	76.44
cg03651886	rs7081974	10.00	80215612	C	T	0.94	-0.82	0.08	3.975E-22	93.54
cg03651886	rs61851127	10.00	80216946	G	T	0.85	-0.60	0.06	6.473E-21	88.02
cg03651886	rs7918709	10.00	80217968	G	A	0.49	0.39	0.06	2.964E-10	39.70
cg03651886	rs10824611	10.00	80218096	C	A	0.85	-0.59	0.06	4.183E-21	88.89
cg03651886	rs11002563	10.00	80218672	T	A	0.86	-0.59	0.06	7.089E-21	87.84
cg03651886	rs16935963	10.00	80219009	T	A	0.94	-0.78	0.09	3.409E-19	80.19
cg03651886	rs16935969	10.00	80220796	A	G	0.94	-0.76	0.09	2.846E-18	75.99
cg03651886	rs2692732	10.00	80221846	C	T	0.53	-0.34	0.04	5.083E-15	61.23
cg03651886	rs2578122	10.00	80222228	G	A	0.53	-0.33	0.04	1.791E-14	58.75
cg03651886	rs2670199	10.00	80222369	T	C	0.53	-0.34	0.04	1.211E-14	59.52
cg03651886	rs2670200	10.00	80222411	A	C	0.53	-0.34	0.04	1.211E-14	59.52
cg03651886	rs10740479	10.00	80222468	G	A	0.86	-0.61	0.06	1.695E-21	90.67
cg03651886	rs2574691	10.00	80222570	T	C	0.53	-0.34	0.04	1.211E-14	59.52
cg03651886	rs2692731	10.00	80222674	A	G	0.53	-0.34	0.04	1.211E-14	59.52
cg03651886	rs11002567	10.00	80223273	T	G	0.86	-0.62	0.06	5.642E-22	92.85
cg03651886	rs11002569	10.00	80223728	G	T	0.76	0.42	0.07	1.116E-09	37.11
cg03651886	rs11002570	10.00	80224320	A	G	0.94	-0.97	0.10	2.458E-22	94.49
cg03651886	rs10824613	10.00	80224329	C	G	0.86	-0.62	0.06	5.674E-22	92.84
cg03651886	rs2570547	10.00	80224364	G	C	0.53	-0.34	0.04	3.094E-15	62.21
cg03651886	rs10824614	10.00	80224591	G	A	0.86	-0.61	0.06	1.130E-21	91.48
cg03651886	rs10762785	10.00	80224705	C	T	0.86	-0.59	0.06	6.369E-20	83.50
cg03651886	rs10824615	10.00	80224729	C	G	0.86	-0.58	0.06	2.718E-19	80.63
cg03651886	rs10762786	10.00	80225164	C	T	0.62	-0.37	0.04	5.195E-17	70.26
cg03651886	rs10762787	10.00	80225251	T	C	0.74	-0.42	0.07	1.439E-09	36.62
cg03651886	rs11002571	10.00	80226280	C	T	0.62	0.39	0.06	5.531E-10	38.48
cg03651886	rs2578109	10.00	80227321	A	G	0.47	-0.35	0.06	2.816E-08	30.83
cg03651886	rs10824616	10.00	80229771	G	C	0.91	-1.82	0.05	5.617E-300	1370.43
cg03651886	rs61851140	10.00	80229872	A	C	0.09	0.99	0.11	4.393E-20	84.23
cg03651886	rs10824617	10.00	80230227	A	G	0.91	-1.84	0.05	2.029E-304	1390.87
cg03651886	rs11597465	10.00	80230590	A	G	0.91	-1.77	0.05	6.223E-301	1374.83
cg03651886	rs11593190	10.00	80230616	C	T	0.91	-1.81	0.05	5.160E-303	1384.40

cg03651886	rs61851141	10.00	80230938	A	G	0.91	-1.81	0.05	4.650E-303	1384.60
cg03651886	rs2570548	10.00	80231107	T	C	0.85	-1.47	0.04	0.000E+00	1654.97
cg03651886	rs2692726	10.00	80231152	A	C	0.85	-1.48	0.04	0.000E+00	1658.69
cg03651886	rs2692725	10.00	80231392	A	G	0.85	-1.47	0.04	0.000E+00	1659.49
cg03651886	rs4979978	10.00	80231605	C	T	0.85	-1.47	0.04	0.000E+00	1707.26
cg03651886	rs4979979	10.00	80231691	G	T	0.85	-1.47	0.04	0.000E+00	1707.79
cg03651886	rs4979816	10.00	80231861	C	A	0.85	-1.48	0.04	0.000E+00	1730.75
cg03651886	rs4979818	10.00	80232093	G	A	0.85	-1.47	0.04	0.000E+00	1707.92
cg03651886	rs4979819	10.00	80232253	G	A	0.85	-1.47	0.04	0.000E+00	1707.92
cg03651886	rs4979820	10.00	80232295	A	G	0.85	-1.47	0.04	0.000E+00	1707.92
cg03651886	rs12251308	10.00	80232518	A	G	0.48	-0.34	0.06	3.744E-08	30.28
cg03651886	rs2574688	10.00	80232743	T	A	0.46	-0.34	0.06	3.744E-08	30.28
cg03651886	rs2574687	10.00	80233061	T	C	0.85	-1.44	0.04	0.000E+00	1592.44
cg03651886	rs2692737	10.00	80233646	A	G	0.85	-1.47	0.04	0.000E+00	1621.85
cg03651886	rs2574686	10.00	80233879	G	C	0.48	-0.34	0.06	3.744E-08	30.28
cg03651886	rs2578110	10.00	80234116	G	T	0.85	-1.44	0.04	0.000E+00	1565.90
cg03651886	rs7088059	10.00	80234154	G	A	0.64	0.43	0.06	2.893E-12	48.76
cg03651886	rs2578111	10.00	80234210	C	T	0.94	-1.06	0.08	1.612E-42	186.77
cg03651886	rs2254238	10.00	80234372	T	C	0.48	-0.34	0.06	3.248E-08	30.55
cg03651886	rs10509397	10.00	80235368	C	G	0.47	-0.43	0.06	1.297E-12	50.33
cg03651886	rs11002575	10.00	80235805	C	G	0.87	-1.56	0.04	0.000E+00	1476.62
cg03651886	rs66483670	10.00	80236268	C	T	0.94	-0.64	0.07	2.485E-19	80.81
cg03651886	rs11817544	10.00	80236999	C	A	0.94	-0.64	0.07	9.675E-20	82.67
cg03651886	rs59483558	10.00	80238015	T	C	0.94	-0.64	0.07	6.379E-20	83.50
cg03651886	rs10509398	10.00	80238142	C	A	0.94	-0.64	0.07	6.379E-20	83.50
cg03651886	rs67020476	10.00	80239089	C	T	0.94	-0.64	0.07	6.141E-20	83.57
cg03651886	rs16935995	10.00	80239425	G	T	0.94	-0.64	0.07	5.484E-20	83.80
cg03651886	rs16935996	10.00	80240206	A	G	0.94	-0.64	0.07	3.733E-20	84.56
cg03651886	rs55635664	10.00	80240400	T	A	0.94	-0.64	0.07	5.912E-20	83.65
cg03651886	rs60101611	10.00	80240493	G	A	0.94	-0.64	0.07	5.912E-20	83.65
cg03651886	rs55923438	10.00	80240560	G	A	0.94	-0.64	0.07	5.912E-20	83.65
cg03651886	rs60427575	10.00	80240584	C	T	0.94	-0.64	0.07	5.912E-20	83.65
cg03651886	rs56121054	10.00	80241087	A	G	0.94	-0.64	0.07	5.912E-20	83.65
cg03651886	rs60579229	10.00	80241148	C	G	0.94	-0.64	0.07	5.912E-20	83.65
cg03651886	rs57880213	10.00	80241226	T	C	0.94	-0.64	0.07	5.912E-20	83.65
cg03651886	rs55952028	10.00	80241270	C	A	0.94	-0.64	0.07	5.912E-20	83.65
cg03651886	rs16935998	10.00	80241364	G	A	0.94	-0.64	0.07	5.912E-20	83.65

cg03651886	rs16935999	10.00	80241411	C	T	0.94	-0.64	0.07	5.912E-20	83.65
cg03651886	rs16936000	10.00	80241583	C	A	0.94	-0.64	0.07	5.912E-20	83.65
cg03651886	rs55804217	10.00	80241627	T	G	0.94	-0.64	0.07	5.912E-20	83.65
cg03651886	rs2840175	10.00	80242036	T	G	0.94	-0.64	0.07	5.912E-20	83.65
cg03651886	rs59575952	10.00	80243039	C	T	0.94	-0.64	0.07	4.434E-20	84.22
cg03651886	rs60429535	10.00	80243101	C	T	0.94	-0.64	0.07	4.434E-20	84.22
cg03651886	rs57486094	10.00	80243786	A	C	0.94	-0.64	0.07	5.990E-20	83.62
cg03651886	rs60261162	10.00	80244345	G	C	0.94	-0.64	0.07	6.687E-20	83.40
cg03651886	rs67185935	10.00	80244589	G	A	0.94	-0.64	0.07	6.687E-20	83.40
cg03651886	rs4554825	10.00	80244623	C	T	0.94	-0.64	0.07	4.398E-20	84.23
cg03651886	rs3928165	10.00	80244968	C	T	0.94	-0.63	0.07	7.883E-20	83.08
cg03651886	rs4391765	10.00	80245052	G	A	0.94	-0.64	0.07	6.687E-20	83.40
cg03651886	rs4295984	10.00	80245475	C	T	0.94	-0.63	0.07	1.612E-19	81.67
cg03651886	rs1019967	10.00	80245744	A	T	0.94	-0.64	0.07	6.687E-20	83.40
cg03651886	rs1019968	10.00	80245747	T	C	0.94	-0.64	0.07	6.687E-20	83.40
cg03651886	rs56188421	10.00	80246987	A	G	0.94	-0.64	0.07	6.687E-20	83.40
cg03651886	rs72810232	10.00	80247265	A	C	0.94	-0.64	0.07	6.687E-20	83.40
cg03651886	rs72810233	10.00	80247277	A	T	0.94	-0.64	0.07	6.687E-20	83.40
cg03651886	rs58616876	10.00	80247845	A	G	0.94	-0.64	0.07	6.687E-20	83.40
cg03651886	rs11813027	10.00	80248110	G	C	0.94	-0.64	0.07	6.687E-20	83.40
cg03651886	rs67792918	10.00	80248804	G	C	0.94	-0.64	0.07	6.687E-20	83.40
cg03651886	rs60323283	10.00	80249385	C	T	0.94	-0.64	0.07	6.687E-20	83.40
cg03651886	rs60183993	10.00	80249582	C	A	0.94	-0.64	0.07	6.687E-20	83.40
cg03651886	rs61392627	10.00	80249725	G	C	0.94	-0.64	0.07	6.687E-20	83.40
cg03651886	rs58722389	10.00	80249827	A	G	0.94	-0.64	0.07	6.687E-20	83.40
cg03651886	rs56076239	10.00	80250710	G	A	0.94	-0.64	0.07	6.687E-20	83.40
cg03651886	rs7918954	10.00	80251721	C	T	0.90	-0.87	0.11	6.801E-15	60.66
cg03651886	rs66949455	10.00	80251780	C	T	0.94	-0.64	0.07	6.944E-20	83.33
cg03651886	rs56002941	10.00	80251977	A	G	0.94	-0.64	0.07	6.944E-20	83.33
cg03651886	rs2163991	10.00	80252664	G	A	0.94	-1.12	0.13	3.589E-19	80.08
cg03651886	rs3997831	10.00	80254088	T	G	0.94	-1.12	0.13	3.589E-19	80.08
cg03651886	rs67884025	10.00	80254475	G	A	0.94	-1.12	0.13	3.589E-19	80.08
cg03651886	rs67451344	10.00	80255661	A	C	0.94	-1.12	0.13	3.589E-19	80.08
cg03651886	rs56231799	10.00	80258348	T	C	0.94	-1.12	0.13	3.589E-19	80.08
cg03651886	rs1821372	10.00	80260588	T	C	0.94	-1.12	0.13	3.589E-19	80.08
cg03651886	rs1821676	10.00	80260703	T	C	0.94	-1.12	0.13	3.589E-19	80.08
cg03651886	rs66623428	10.00	80261767	C	T	0.94	-1.08	0.12	4.198E-18	75.23

cg03651886	rs55889428	10.00	80266092	C	T	0.94	-1.12	0.13	3.589E-19	80.08
cg03651886	rs1473872	10.00	80270499	A	T	0.96	-1.07	0.14	3.922E-14	57.21
cg03651886	rs56053227	10.00	80286577	G	A	0.96	-0.95	0.14	4.367E-12	47.95
cg03651886	rs72810250	10.00	80288883	G	A	0.96	-0.95	0.14	4.367E-12	47.95
cg03651886	rs72810251	10.00	80288910	G	A	0.96	-0.95	0.14	4.367E-12	47.95
cg03651886	rs55750209	10.00	80290721	G	A	0.96	-0.95	0.14	4.367E-12	47.95
cg03651886	rs56174913	10.00	80292275	C	T	0.96	-0.95	0.14	4.367E-12	47.95
cg03651886	rs2593153	10.00	80302788	G	C	0.95	-0.92	0.14	2.304E-11	44.69
cg03651886	rs16936045	10.00	80303549	G	C	0.96	-0.92	0.14	3.556E-11	43.84
cg03651886	rs56390301	10.00	80309274	A	G	0.96	-0.92	0.14	3.556E-11	43.84
cg03651886	rs12219399	10.00	80310047	G	A	0.95	-0.92	0.14	3.556E-11	43.84
cg03651886	rs7090749	10.00	80310636	A	G	0.95	-0.90	0.14	3.393E-11	43.94
cg03651886	rs10509399	10.00	80312035	T	C	0.96	-0.95	0.13	2.014E-12	49.47
cg03651886	rs34269041	10.00	80312095	G	A	0.94	-0.99	0.13	1.654E-13	54.38
cg03651886	rs2019432	10.00	80312407	A	G	0.95	-0.95	0.13	2.014E-12	49.47
cg03651886	rs2593150	10.00	80312891	T	C	0.95	-0.99	0.13	2.247E-13	53.78
cg03651886	rs1369754	10.00	80314300	C	T	0.95	-0.99	0.13	2.247E-13	53.78
cg03651886	rs2007065	10.00	80314859	A	G	0.95	-0.99	0.13	2.247E-13	53.78
cg03651886	rs55983992	10.00	80315902	C	T	0.96	-0.99	0.13	2.247E-13	53.78
cg03651886	rs16936070	10.00	80316027	C	A	0.96	-0.99	0.13	2.247E-13	53.78
cg03651886	rs987279	10.00	80317287	C	T	0.95	-0.99	0.13	1.261E-13	54.91
cg03651886	rs16936074	10.00	80317669	A	T	0.96	-0.99	0.13	1.261E-13	54.91
cg03651886	rs1368765	10.00	80318719	G	A	0.95	-0.84	0.13	3.480E-11	43.89
cg03651886	rs11002603	10.00	80318894	A	C	0.95	-0.98	0.13	1.261E-13	54.91
cg03651886	rs72810277	10.00	80319590	G	T	0.95	-0.99	0.13	1.261E-13	54.91
cg03651886	rs12244473	10.00	80320754	T	G	0.95	-0.99	0.13	1.261E-13	54.91
cg03651886	rs72810278	10.00	80320755	C	T	0.96	-0.99	0.13	1.261E-13	54.91
cg03651886	rs986817	10.00	80320921	A	G	0.95	-0.98	0.13	2.247E-13	53.78
cg03651886	rs12253312	10.00	80321424	A	G	0.95	-0.98	0.13	2.247E-13	53.78
cg03651886	rs59916858	10.00	80321707	G	A	0.96	-0.98	0.13	4.057E-13	52.62
cg03651886	rs12248249	10.00	80322099	T	G	0.95	-0.98	0.13	3.162E-13	53.11
cg03651886	rs4979990	10.00	80322669	T	C	0.95	-0.98	0.13	3.162E-13	53.11
cg03651886	rs12257281	10.00	80323206	A	T	0.95	-0.98	0.13	3.162E-13	53.11
cg03986574	rs12417551	11.00	64447026	G	A	0.92	-0.59	0.10	2.611E-09	35.45
cg03986574	rs11231853	11.00	64450479	C	G	0.92	-0.59	0.10	2.611E-09	35.45
cg03986574	rs12575704	11.00	64450961	G	A	0.92	-0.59	0.10	2.611E-09	35.45
cg03986574	rs72643567	11.00	64465514	C	T	0.92	-0.59	0.10	4.256E-09	34.50

cg03986574	rs72643568	11.00	64472977	C	T	0.92	-0.60	0.10	1.465E-09	36.58
cg03986574	rs3809076	11.00	64482546	G	A	0.92	-0.65	0.10	8.546E-11	42.13
cg03986574	rs496914	11.00	64487394	T	C	0.91	-0.63	0.10	5.872E-10	38.36
cg03986574	rs11231859	11.00	64488054	G	A	0.92	-0.60	0.10	2.371E-09	35.64
cg03986574	rs58483071	11.00	64488291	G	A	0.92	-0.61	0.10	2.371E-09	35.64
cg03986574	rs2375335	11.00	64490123	A	G	0.91	-0.61	0.10	2.371E-09	35.64
cg03986574	rs693235	11.00	64492762	C	A	0.91	-0.59	0.10	5.288E-09	34.08
cg03986574	rs2267918	11.00	64495284	T	C	0.91	-0.61	0.10	2.371E-09	35.64
cg03986574	rs2230414	11.00	64496357	G	T	0.92	-0.61	0.10	2.034E-09	35.94
cg03986574	rs2003293	11.00	64496997	C	T	0.92	-0.61	0.10	2.034E-09	35.94
cg03986574	rs494128	11.00	64498625	A	G	0.91	-0.59	0.10	1.345E-08	32.26
cg03986574	rs580374	11.00	64499482	C	T	0.91	-0.61	0.10	2.034E-09	35.94
cg03986574	rs1207181	11.00	64500411	G	A	0.91	-0.61	0.10	2.034E-09	35.94
cg03986574	rs2284301	11.00	64501991	C	T	0.92	-0.62	0.10	1.485E-09	36.55
cg03986574	rs10897524	11.00	64503363	G	A	0.92	-0.63	0.10	6.441E-10	38.18
cg03986574	rs667237	11.00	64506539	A	G	0.91	-0.63	0.10	6.441E-10	38.18
cg03986574	rs2073797	11.00	64511050	C	G	0.92	-0.60	0.11	2.651E-08	30.95
cg03986574	rs2073798	11.00	64511322	G	T	0.92	-0.59	0.11	4.860E-08	29.77
cg03986574	rs12223914	11.00	64511886	G	T	0.93	-0.60	0.11	2.651E-08	30.95
cg03986574	rs569602	11.00	64514506	A	G	0.93	-0.60	0.11	2.651E-08	30.95
cg03986574	rs555974	11.00	64516477	G	T	0.92	-0.60	0.11	2.651E-08	30.95
cg03986574	rs1207113	11.00	64517047	A	G	0.92	-0.60	0.11	2.651E-08	30.95
cg03986574	rs532747	11.00	64519062	T	C	0.93	-0.60	0.11	2.651E-08	30.95
cg03986574	rs686171	11.00	64519345	G	A	0.93	-0.62	0.11	1.093E-08	32.67
cg03986574	rs2959652	11.00	64520942	T	G	0.91	-0.67	0.10	5.697E-11	42.92
cg03986574	rs625172	11.00	64522066	G	A	0.92	-0.62	0.11	1.093E-08	32.67
cg03986574	rs547066	11.00	64523494	C	A	0.93	-0.62	0.11	9.396E-09	32.96
cg03986574	rs7938455	11.00	64524781	C	T	0.93	-0.62	0.11	1.093E-08	32.67
cg03986574	rs630966	11.00	64524911	G	C	0.92	-0.34	0.06	2.944E-09	35.22
cg03986574	rs589691	11.00	64525216	T	C	0.91	-0.67	0.10	2.922E-11	44.23
cg03986574	rs490980	11.00	64525464	T	C	0.91	-0.67	0.10	2.922E-11	44.23
cg03986574	rs489192	11.00	64525644	T	G	0.92	-0.65	0.11	9.070E-10	37.52
cg03986574	rs477549	11.00	64527080	T	C	0.91	-0.48	0.06	2.789E-17	71.49
cg03986574	rs483962	11.00	64527751	A	G	0.91	-0.51	0.06	8.462E-19	78.39
cg03986574	rs637332	11.00	64528750	A	G	0.91	-0.52	0.06	2.444E-19	80.84
cg03986574	rs620006	11.00	64530431	A	G	0.91	-0.68	0.10	1.560E-11	45.46
cg03986574	rs610637	11.00	64530709	G	A	0.96	-0.61	0.11	7.646E-08	28.89

cg03986574	rs523200	11.00	64532579	A	C	0.91	-0.68	0.10	1.560E-11	45.46
cg03986574	rs680273	11.00	64539635	C	G	0.91	-0.67	0.10	2.790E-11	44.32
cg03986574	rs633923	11.00	64542272	C	A	0.91	-0.51	0.06	6.486E-19	78.91
cg03986574	rs474707	11.00	64544135	C	T	0.91	-0.52	0.06	2.466E-19	80.83
cg03986574	rs7111870	11.00	64544720	G	A	0.01	-0.62	0.11	5.824E-09	33.89
cg03986574	rs3741398	11.00	64546106	C	T	0.92	-0.55	0.07	9.904E-16	64.45
cg03986574	rs1633462	11.00	64546257	C	G	0.92	-0.50	0.07	1.403E-13	54.70
cg03986574	rs606458	11.00	64546391	T	C	0.91	-0.68	0.10	1.560E-11	45.46
cg03986574	rs487105	11.00	64546891	G	A	0.92	-0.50	0.07	1.431E-13	54.66
cg03986574	rs593394	11.00	64546991	C	T	0.92	-0.50	0.07	1.431E-13	54.66
cg03986574	rs673224	11.00	64548015	A	G	0.92	-0.63	0.11	3.407E-09	34.94
cg03986574	rs684720	11.00	64548429	C	G	0.91	-0.50	0.07	1.431E-13	54.66
cg03986574	rs683841	11.00	64548581	G	T	0.91	-0.54	0.07	3.239E-15	62.12
cg03986574	rs523752	11.00	64548584	A	G	0.90	-0.68	0.10	1.560E-11	45.46
cg03986574	rs674297	11.00	64549514	C	T	0.92	-0.50	0.07	1.431E-13	54.66
cg03986574	rs607389	11.00	64553578	A	T	0.91	-0.44	0.06	3.015E-14	57.72
cg03986574	rs533447	11.00	64561908	C	T	0.92	-0.68	0.10	1.560E-11	45.46
cg03986574	rs677298	11.00	64564208	A	G	0.92	-0.68	0.10	1.560E-11	45.46
cg03986574	rs582980	11.00	64565240	C	T	0.92	-0.68	0.10	1.560E-11	45.46
cg03986574	rs1143937	11.00	64569000	C	T	0.92	-0.65	0.10	4.791E-10	38.76
cg03986574	rs669976	11.00	64573589	T	C	0.92	-0.65	0.10	4.791E-10	38.76
cg03986574	rs67808744	11.00	64576598	C	T	0.92	-0.64	0.11	9.752E-10	37.37
cg03986574	rs624975	11.00	64576954	C	T	0.92	-0.64	0.11	9.752E-10	37.37
cg03986574	rs509606	11.00	64577620	G	C	0.92	-0.64	0.10	9.752E-10	37.37
cg03986574	rs675254	11.00	64581570	T	A	0.94	-0.61	0.11	2.145E-08	31.36
cg03986574	rs600347	11.00	64581696	A	G	0.93	-0.61	0.11	1.155E-08	32.56
cg03986574	rs524386	11.00	64584959	T	C	0.93	-0.58	0.11	4.382E-08	29.97
cg06880857	rs73331532	14.00	95594272	C	A	0.10	-0.53	0.10	8.196E-08	28.76
cg24612305	rs4257260	17.00	17857003	A	G	0.56	-0.34	0.04	4.196E-15	61.61
cg24612305	rs4553680	17.00	17857722	A	G	0.55	-0.31	0.04	3.709E-12	48.27
cg24612305	rs4413022	17.00	17858280	G	C	0.56	-0.34	0.04	4.211E-15	61.60
cg24612305	rs8065970	17.00	17858771	A	G	0.56	-0.34	0.04	4.196E-15	61.61
cg24612305	rs4414547	17.00	17859052	G	T	0.56	-0.34	0.04	4.196E-15	61.61
cg24612305	rs8070748	17.00	17859421	A	G	0.56	-0.34	0.04	4.196E-15	61.61
cg24612305	rs8077338	17.00	17860546	T	C	0.51	-0.33	0.05	6.201E-13	51.78
cg24612305	rs4616340	17.00	17861360	T	C	0.56	-0.34	0.04	4.213E-15	61.60
cg24612305	rs8080823	17.00	17864048	T	C	0.55	-0.31	0.04	3.709E-12	48.27

cg24612305	rs12603148	17.00	17865257	C	T	0.56	-0.39	0.04	9.888E-19	78.08
cg24612305	rs9905284	17.00	17865752	G	A	0.56	-0.34	0.04	4.198E-15	61.60
cg24612305	rs10048206	17.00	17866897	A	G	0.56	-0.34	0.04	5.717E-15	61.00
cg24612305	rs12600546	17.00	17867614	A	G	0.56	-0.34	0.04	3.606E-15	61.90
cg24612305	rs7213225	17.00	17869111	T	C	0.56	-0.34	0.04	3.606E-15	61.90
cg24612305	rs6502629	17.00	17869642	A	G	0.51	-0.32	0.04	7.482E-13	51.41
cg24612305	rs9635697	17.00	17870394	T	A	0.56	-0.34	0.04	3.606E-15	61.90
cg24612305	rs60073596	17.00	17870974	G	T	0.62	-0.28	0.05	6.069E-09	33.81
cg24612305	rs9908832	17.00	17871068	C	T	0.56	-0.39	0.04	9.888E-19	78.08
cg24612305	rs9635698	17.00	17871884	C	T	0.56	-0.34	0.04	3.606E-15	61.90
cg24612305	rs4343339	17.00	17872589	T	C	0.56	-0.34	0.04	3.445E-15	61.99
cg24612305	rs8068175	17.00	17874486	C	T	0.60	-0.36	0.04	1.206E-15	64.06
cg24612305	rs57728924	17.00	17875342	A	G	0.60	-0.36	0.04	5.327E-16	65.67
cg24612305	rs59304093	17.00	17875407	A	G	0.60	-0.36	0.04	5.327E-16	65.67
cg24612305	rs9914127	17.00	17875836	A	T	0.61	-0.45	0.04	9.470E-29	123.77
cg24612305	rs11078408	17.00	17876011	A	G	0.61	-0.45	0.04	9.470E-29	123.77
cg24612305	rs11078409	17.00	17876296	C	G	0.61	-0.45	0.04	1.080E-28	123.51
cg24612305	rs11870660	17.00	17877096	C	T	0.60	-0.36	0.04	5.006E-16	65.79
cg24612305	rs4471742	17.00	17877771	C	T	0.61	-0.45	0.04	1.080E-28	123.51
cg24612305	rs4299203	17.00	17878159	T	G	0.60	-0.36	0.04	5.296E-16	65.68
cg24612305	rs4321247	17.00	17878612	A	G	0.56	-0.31	0.05	3.137E-12	48.60
cg24612305	rs7219320	17.00	17880877	A	G	0.60	-0.36	0.04	3.882E-16	66.30
cg24612305	rs3935505	17.00	17882020	T	G	0.70	-0.36	0.05	1.385E-15	63.79
cg24612305	rs3935506	17.00	17882059	G	A	0.70	-0.43	0.05	1.926E-20	85.87
cg24612305	rs4635395	17.00	17882087	G	A	0.70	-0.43	0.05	1.846E-20	85.95
cg24612305	rs11871231	17.00	17883848	A	G	0.64	-0.36	0.05	2.225E-15	62.85
cg24612305	rs11869491	17.00	17884055	T	C	0.64	-0.35	0.04	1.111E-14	59.69
cg24612305	rs4072738	17.00	17884547	A	C	0.63	-0.35	0.04	1.581E-14	58.99
cg24612305	rs4072739	17.00	17884660	G	A	0.63	-0.35	0.04	1.582E-14	58.99
cg24612305	rs4925133	17.00	17884801	A	G	0.60	-0.34	0.04	5.725E-14	56.46
cg24612305	rs4459604	17.00	17888549	T	C	0.62	-0.35	0.04	5.720E-15	61.00
cg24612305	rs7207821	17.00	17891781	A	G	0.62	-0.44	0.04	1.437E-26	113.81
cg24612305	rs62072048	17.00	17894750	G	A	0.62	-0.44	0.04	3.780E-27	116.45
cg24612305	rs4368210	17.00	17896090	C	T	0.62	-0.40	0.06	7.926E-13	51.30
cg24612305	rs4584886	17.00	17896205	C	T	0.62	-0.40	0.06	7.952E-13	51.29
cg24612305	rs4506969	17.00	17899839	A	G	0.62	-0.40	0.06	7.541E-13	51.40
cg24612305	rs28537385	17.00	17902135	G	A	0.62	-0.40	0.06	7.926E-13	51.30

cg24612305	rs62072050	17.00	17906564	C	G	0.62	-0.40	0.06	7.926E-13	51.30
cg24612305	rs9896837	17.00	17924868	A	G	0.62	-0.40	0.06	1.046E-12	50.76
cg24612305	rs7215524	17.00	17926605	C	T	0.62	-0.40	0.06	8.133E-13	51.25
cg24612305	rs2955378	17.00	17930253	G	A	0.62	-0.40	0.06	8.101E-13	51.26
cg24612305	rs2955377	17.00	17932818	G	A	0.62	-0.40	0.06	8.133E-13	51.25
cg24612305	rs7224047	17.00	17932931	G	A	0.62	-0.40	0.06	8.133E-13	51.25
cg24612305	rs2955385	17.00	17939247	C	T	0.62	-0.40	0.06	8.503E-13	51.16
cg24612305	rs2955384	17.00	17939573	G	A	0.62	-0.40	0.06	8.503E-13	51.16
cg24612305	rs7503738	17.00	17940305	A	T	0.62	-0.40	0.06	8.503E-13	51.16
cg24612305	rs6502633	17.00	17941037	G	C	0.62	-0.40	0.06	8.503E-13	51.16
cg24612305	rs7406982	17.00	17942613	C	T	0.62	-0.40	0.06	7.692E-13	51.36
cg24612305	rs2955380	17.00	17944349	G	A	0.62	-0.40	0.06	7.692E-13	51.36
cg24612305	rs2955381	17.00	17945500	C	G	0.62	-0.40	0.06	1.070E-12	50.71
cg24612305	rs2955359	17.00	17946730	C	T	0.62	-0.40	0.06	1.070E-12	50.71
cg24612305	rs2955354	17.00	17948716	C	G	0.62	-0.40	0.06	1.070E-12	50.71
cg24612305	rs2955353	17.00	17948979	C	T	0.62	-0.40	0.06	1.070E-12	50.71
cg24612305	rs7207461	17.00	17949789	G	T	0.62	-0.40	0.06	7.416E-13	51.43
cg24612305	rs12940282	17.00	17950001	C	T	0.62	-0.40	0.06	7.416E-13	51.43
cg24612305	rs8070731	17.00	17952447	C	T	0.62	-0.40	0.06	7.392E-13	51.44
cg24612305	rs4925135	17.00	17952868	C	T	0.62	-0.40	0.06	7.380E-13	51.44
cg24612305	rs2955350	17.00	17953548	A	G	0.62	-0.40	0.06	7.380E-13	51.44
cg24612305	rs8080602	17.00	17954728	C	G	0.62	-0.40	0.06	7.380E-13	51.44
cg24612305	rs8080334	17.00	17954764	G	A	0.62	-0.40	0.06	7.234E-13	51.48
cg24612305	rs6502634	17.00	17955344	C	T	0.62	-0.40	0.06	7.380E-13	51.44
cg24612305	rs11658257	17.00	17956459	C	G	0.62	-0.40	0.06	7.392E-13	51.44
cg24612305	rs2955352	17.00	17961389	C	T	0.62	-0.40	0.06	7.404E-13	51.43
cg24612305	rs2955369	17.00	17961407	A	C	0.62	-0.40	0.06	7.404E-13	51.43
cg10664184	rs10409801	19.00	17407415	G	A	0.88	0.54	0.09	3.194E-09	35.06
cg10664184	rs2303745	19.00	17420289	T	G	0.87	0.63	0.10	5.254E-11	43.08

ST-7: the IVs of cpG sites. EA, effect allele. NEA, non-effect allele. MAF, minor allele frequency. F, F-statistic, is valued by (Beta/SE)^2.

Outcome	Exposure	Methods	IVs	p.value	OR	95%LCI	95%UCI	$p_{heterogeneity}$	$p_{pleiotropy}$
Depression	cg09566392	IVW radial	26	1.05E-08	0.9951	0.9934	0.9967	>0.999	0.699
		Simple median	26	4.57E-01	0.9946	0.9806	1.0088		
		Maximum likelihood	26	3.84E-01	0.9951	0.9840	1.0062		
Depression	cg19565299	IVW radial	13	1.04E-05	1.0071	1.0039	1.0103	0.999	0.266
		Simple median	13	4.92E-02	1.0098	1.0000	1.0196		
		Maximum likelihood	13	7.45E-02	1.0072	0.9993	1.0151		
Depression	cg17054900	IVW radial	14	1.00E-125	0.9924	0.9923	0.9925	>0.999	0.99
		Simple median	14	1.57E-01	0.9924	0.9820	1.0029		
		Maximum likelihood	14	9.07E-02	0.9924	0.9837	1.0012		
Depression	cg00195828	IVW radial	2	4.55E-01	0.9908	0.9671	1.0151	0.626	NA
		Simple median	NA	NA	NA	NA	NA		
		Maximum likelihood	2	7.16E-01	0.9908	0.9426	1.0414		
Depression	cg03651886	IVW radial	151	1.37E-54	1.0294	1.0257	1.0332	5.40E-07	0.086
		Simple median	151	7.85E-49	1.0480	1.0415	1.0546		
		Maximum likelihood	151	2.00E-86	1.0296	1.0267	1.0326		
Depression	cg03986574	IVW radial	63	1.00E-158	1.0276	1.0263	1.0289	>0.999	0.24
		Simple median	63	6.53E-14	1.0276	1.0203	1.0349		
		Maximum likelihood	63	1.50E-20	1.0276	1.0217	1.0336		
Depression	cg06880857	IVW radial	NA	NA	NA	NA	NA	>0.999	0.169
		Simple median	NA	NA	NA	NA	NA		
		Maximum likelihood	NA	NA	NA	NA	NA		
Depression	cg24612305	IVW radial	68	5.24E-05	0.9949	0.9924	0.9973	0.843	0.169
		Simple median	68	6.70E-01	0.9985	0.9916	1.0054		
		Maximum likelihood	68	6.23E-02	0.9948	0.9895	1.0003		
Depression	cg10664184	IVW radial	2	1.29E-41	0.9547	0.9483	0.9611	0.843	NA
		Simple median	NA	NA	NA	NA	NA		
		Maximum likelihood	2	1.05E-02	0.9547	0.9214	0.9892		

ST-8: Mendelian randomization results by IVW radial, weight median, Simple median and Maximum likelihood. 95%LCI, the lower limit of 95% CI; 95%UCI, the upper limit of 95% CI.

SF-1. The leave-one-out analysis of MR results.

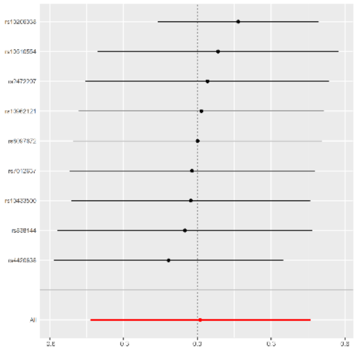

MR leave-one-out sensitivity analysis for "the relative intake of carbohydrate" on "major depression disorder"

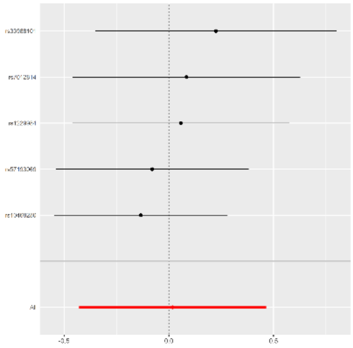

MR leave-one-out sensitivity analysis for "the relative intake of fat" on "major depression disorder"

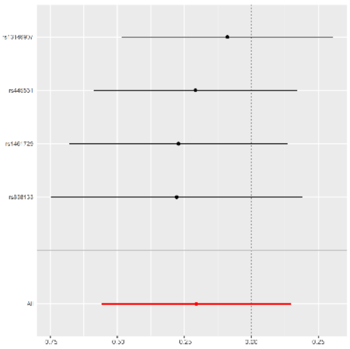

MR leave-one-out sensitivity analysis for "the relative intake of protein" on "major depression disorder"

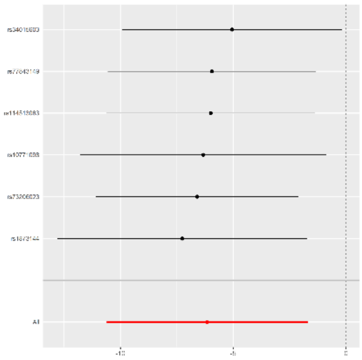

MR leave-one-out sensitivity analysis for "folate" on "major depression disorder"

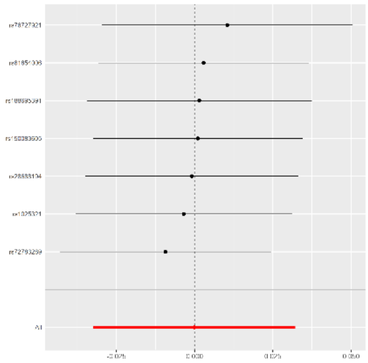

MR leave-one-out sensitivity analysis for "Vitamin B6" on "major depression disorder"

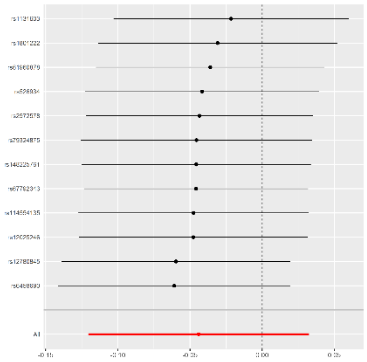

MR leave-one-out sensitivity analysis for "Vitamin B12" on "major depression disorder"

SF-2. The scatter analysis of MR results.

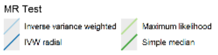

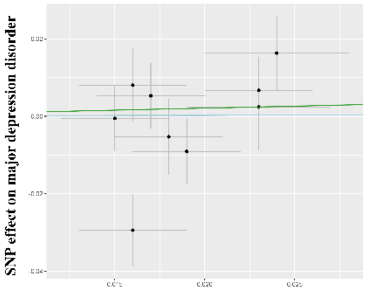

SNP effect on the relative intake of carbohydrate

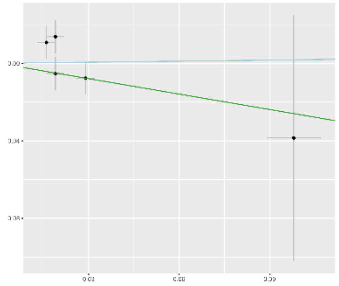

SNP effect on the relative intake of fat

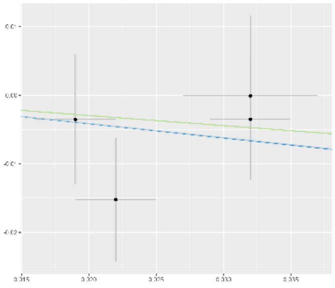

SNP effect on the relative intake of protein

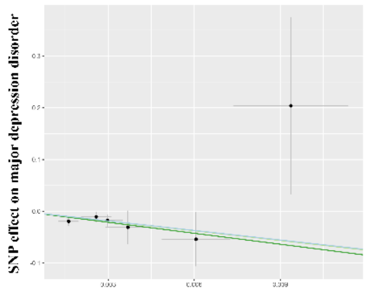

SNP effect on the folate

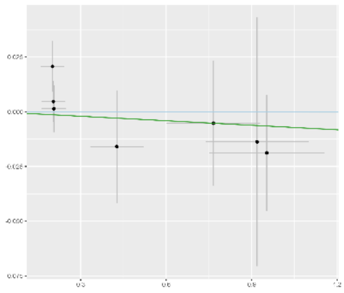

SNP effect on the Vitamin B6

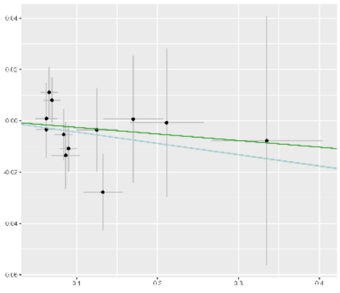

SNP effect on the Vitamin B12

SF-3. The Radial MR results.

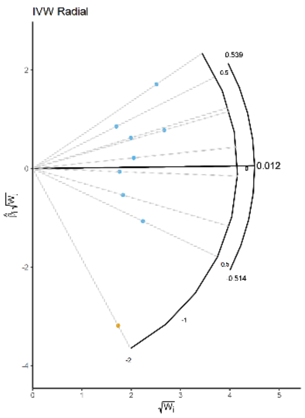

Radial result: the effect of the relative intake of carbohydrate on major depression disorder

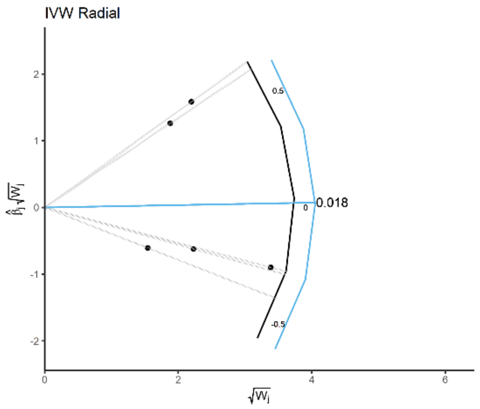

Radial result: the effect of the relative intake of fat on major depression disorder

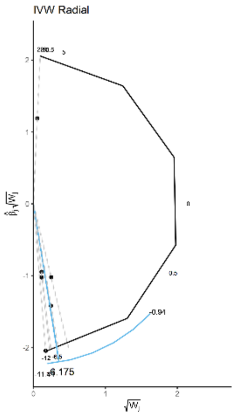

Radial result: the effect of folate on major depression disorder

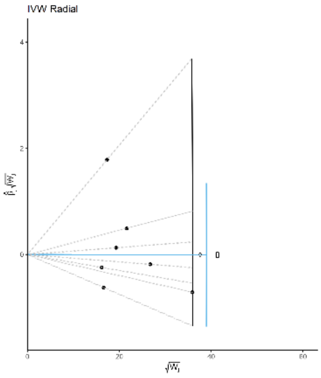

Radial result: the effect of Vitamin B6 on major depression disorder

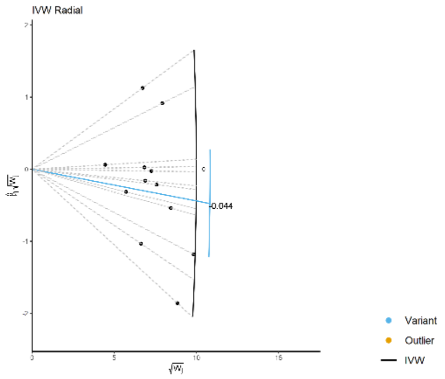

Radial result: the effect of Vitamin B12 on major depression disorder
